# Complete Genome Sequences of T1-Like Phages JMPW1 and JMPW2

**DOI:** 10.1128/genomeA.00601-16

**Published:** 2016-06-23

**Authors:** Mengyu Shen, Hongbin Zhu, Shuguang Lu, Shuai Le, Gang Li, Yinling Tan, Xia Zhao, Wei Shen, Fuquan Hu, Jing Wang

**Affiliations:** Department of Microbiology, College of Basic Medical Science, Third Military Medical University, Chongqing, People’s Republic of China

## Abstract

We report the complete genome sequences of phages JMPW1 (49,840 bp) and JMPW2 (50,298 bp), two T1-like *Escherichia coli* phages isolated from contaminated experiment samples. Although the genomes of JMPW1 and JMPW2 share high identity with T1, they show some differences, which are mainly located in several genes with unknown functions and genes encoding tail fiber proteins and endonucleases.

## GENOME ANNOUNCEMENT

From the 1940s, phages, especially *Escherichia coli* phages, were used as tools to answer basic molecular and biophysical questions (1, 2). Enterobacteria phage T1, a member of the family *Siphoviridae*, was discovered by Delbrück in 1942 (3). Among the T-phage group, studies of phage T1 are rare; the first paper reporting the complete annotated sequence of the T1 genome was published in 2004 (4). To date, only four genome sequences of T1-like phages have been released in GenBank. Here, we present the complete sequences of two novel members of the T1 family, which will extend our knowledge of the T1 family.

*Escherichia* phages JMPW1 and JMPW2 were originally isolated from contaminated experiment samples by the double-layer agar plaque method. Phage particles were purified from bacteria lysates using the standard PEG (polyethylene glycol) protocol, and phage DNA was extracted from the purified phage particles using the SDS-proteinase K protocol (5). The whole-genome sequencing of *Escherichia* phages JMPW1 and JMPW2 was performed using the Ion Torrent PGM at the Academy of Military Medical Sciences (Beijing, China). Sequencing reads with ~1,600-fold (JMPW1) and ~1,900-fold (JMPW2) average coverage were assembled using the Newbler software (version 2.9). Genome annotations of the phages were revealed using fgenesV (http://linux1.softberry.com/berry.phtml?topic=virus&group=programs&subgroup=gfindv) and manually verified by screening all the predicted proteins against the NCBI protein database using BLASTp (6). Then, the results were submitted via the software Sequin (version 13.70) (7).

JMPW1 and JMPW2 are all linear double-stranded DNA phages with genome sizes of 49,840 bp and 50,298 bp, respectively. The two phages have a G+C content of 45.6% (JMPW1) and 45.4% (JMPW2). The genomes contain 76 putative open reading frames (ORFs) (JMPW1) and 82 putative ORFs (JMPW2). No tRNAs were found in the two genomes. JMPW1 and JMPW2 are closely related to enterobacteria phage T1, and both of them share over 90% sequence identity with T1. Nonetheless, the tail fiber proteins of JMPW1 and JMPW2 are very different from T1. Furthermore, compared to T1, function-unknown genes 003, 007, 010, 011, and 012 of JMPW1 and genes 006, 010, 011, 080, 081, and 082 of JMPW2 were added, and two genes related to endonucleases were lost in the JMPW1 genome. Studies of phages JMPW1 and JMPW2 will provide new insight into the diversity of *E. coli* phages.

### Nucleotide sequence accession numbers.

The complete genome sequences of these two phages have been deposited in GenBank under the accession no. KU194206 (JMPW1) and KU194205 (JMPW2).
